# Prevalence and economic evaluation of acute uncomplicated cystitis in women from Japan: a retrospective cohort study

**DOI:** 10.1093/jacamr/dlaf178

**Published:** 2025-10-17

**Authors:** Madison T Preib, Maia R Emden, Naomi C Sacks, Fanny S Mitrani-Gold, Shinyoung Ju, Yoshiaki Kawano, Shinya Kawamatsu, Ashish V Joshi

**Affiliations:** Global Real World Evidence and Health Outcomes, GSK, Collegeville, PA, USA; PRECISIONheor, Boston, MA, USA; HEORStrategies, A Division of ToxStrategies, Boston, MA, USA; Global Real World Evidence and Health Outcomes, GSK, Collegeville, PA, USA; Real World Data Strategy and Partnerships, GSK, London, UK; GSK, Tokyo, Japan; GSK, Tokyo, Japan; Global Real World Evidence and Health Outcomes, GSK, Collegeville, PA, USA

## Abstract

**Background:**

Although Japanese guidelines recommend fluoroquinolones (FQs) and cephalosporins for AUC (acute uncomplicated cystitis) treatment, the emergence of FQ-resistant uropathogens and inappropriate use of antibiotics may lead to treatment failure (TF), and increased healthcare costs. There is a need to understand the epidemiology, treatment patterns, and healthcare cost implications associated with AUC in Japan.

**Methods:**

This retrospective cohort study used the Japanese Medical Database Centre database (1 October 2015–30 November 2021). Female patients (≥18 years) had an AUC diagnosis in the same month as ≥1 oral antibiotic prescription claim in the outpatient setting. The population was stratified into cohorts by TF status and history of AUC recurrence prior to index (pre-index recurrence). Baseline demographics were evaluated in the 12-month pre-index period and age standardized prevalence of AUC was calculated. Treatment patterns and AUC-related costs [2022 Japanese Yen (¥)], were reported for the post-index follow-up period.

**Results:**

Of 71 476 total patients, 62.46% were aged <50 years. Patients had evidence of TF (*n* = 3742; 5.24%) and pre-index recurrence (*n* = 3206; 4.49%). The age standardized prevalence of AUC (2016–2021) decreased from 8.62% to 6.02%, with up to 3.9 million women affected each year. A high proportion of patients with TF received FQs (45.48%) and third generation cephalosporins (43.37%). The mean total AUC-related costs were ¥14 905 and pharmacy costs were ¥1059 per patient, per index AUC episode.

**Conclusion:**

Healthcare providers should consider the cost implications for patients with antibiotic TF or a history of AUC recurrence when selecting antibiotics for empiric treatment in Japan.

## Introduction

Urinary tract infections (UTIs) are among the most common bacterial diseases, with a lifetime incidence of 50%–60% in adult women.^1^ The annual incidence is estimated at 12.6% for females aged ≥18, with increased frequency of infection among younger, sexually active women, and those over 60 years.^1,2^ Approximately 20%–30% of women who have a UTI will have a recurrent UTI, defined as three acute episodes in the previous 12 months or two episodes in the previous 6 months.^3,4^

AUC (acute uncomplicated cystitis) is a lower UTI, which is commonly treated empirically with oral antibiotics and it is characterized by acute symptoms of urgency, dysuria, frequency, and suprapubic pain.^5^ European and US guidelines recommend antibacterials such as sulfamethoxazole-trimethoprim combination, fosfomycin, nitrofurantoin, and pivmecillinam for the treatment of AUC.^6,7^ However, nitrofurantoin and pivmecillinam are not available in Japan.^8–10^ Therefore, the Japanese Association for Infectious Disease (JAID) and the Japanese Society of Chemotherapy (JSC) guidelines jointly recommend fluoroquinolones [FQs (levofloxacin, ciprofloxacin, and tosufloxacin)] as first-line therapy for pre-menopausal women with AUC, and second and third generation cephalosporins and penicillin/beta-lactam inhibitor combinations as first-line therapy for post-menopausal women with AUC.^9^

While antibiotics are effective for the treatment of UTIs such as AUC, inappropriate use of antibiotics may lead to the emergence of antimicrobial resistance (AMR) and treatment failure (TF).^11,12^ TF and inappropriate/suboptimal prescriptions are associated with increased healthcare costs compared with appropriate and optimal antibiotic prescriptions among female outpatients with uncomplicated UTI.^13,14^ A retrospective US cohort study, conducted using Optum Clinformatics Data Mart linked to Premier Healthcare Database, reported that in a population of 5870 patients with uncomplicated UTI who had been prescribed antibiotics, 47.1% had inappropriate/suboptimal prescriptions, which were associated with higher total mean healthcare costs per patient, compared with healthcare costs for patients prescribed appropriate and optimal prescriptions.^13^ A subsequent retrospective cohort study using the US Optum Clinformatics Data Mart further reported that uncomplicated UTI-related inpatient and outpatient costs were higher for patients with versus without TF.^14^ These data highlight the importance of appropriate treatment selection to reduce TF and healthcare cost burden.

An observational study of Japanese claims data over the period of 2013–2016 reported a total of 58 380 antimicrobials were administered to patients with AUC, ∼90% of which were FQs (53%) and third-generation cephalosporins (37%).^15^ These treatments are in line with guidelines;^9^ however, with the emergence of FQ-resistant *Escherichia coli* (*E. coli*), the use of FQs may not only exacerbate AMR but also lead to TF.^15–17^ There remains an unmet need to more fully understand the epidemiology, treatment patterns, and healthcare cost implications associated with AUC in Japan.

This study used claims data to characterize patients with AUC, estimate the annual prevalence of AUC, and assess treatment patterns and costs of treating patients with AUC in Japan.

## Materials and methods

### Study design and objectives

This retrospective cohort study was conducted using the Japanese Medical Database Centre (JMDC) payer claims database from 1 October 2015 to 30 November 2021. The JMDC database contains integrated medical and pharmacy claims for subscribers with union administered health insurance.

The objectives of the study were to characterize demographic and clinical characteristics of female patients with AUC, estimate the annual AUC prevalence, understand treatment patterns, including TF, and quantify costs of treating female patients with AUC in Japan.

Study design is shown in Figure S1 (available as Supplementary data at *JAC-AMR* Online); key study definitions are shown in Table S1. Briefly, the index date was defined as the date of the oral antibiotic prescription for AUC within the same month as an AUC diagnosis. Since medical claims in the JMDC only contain the month and year, the exact date of AUC diagnosis could not be determined; thus, the index date was anchored to the prescription date. The index AUC episode was defined as the 28 days from index date, and if TF was observed, the episode was extended an additional 28 days from the date of TF. TF was defined as any of the following (i) intravenous (IV) antibiotics within 28 days of the index date; (ii) a second oral antibiotic prescription claim (receiving a different antibiotic from initial treatment; absence of a respiratory infection code) within 28 days of the index date; and/or (iii) a second AUC diagnosis in an acute care setting [emergency room (ER)] or inpatient) in the month following the index diagnosis month. AUC recurrence was classified as ≥2 AUC episodes within 6 months or ≥3 AUC episodes within 12 months, including the index AUC (episodes were required to be ≥2 months apart given the limitations of the JMDC data).

### Eligibility criteria

Eligible patients were female, aged ≥18 years with an AUC diagnosis in the same month as ≥1 oral antibiotic prescription claim and had ≥1 claim with a primary or secondary diagnosis code for AUC [based on International Classification of Diseases 10th Revision (ICD-10), Clinical Modification: N300, N309, N390] in the outpatient setting (including ER). Patients had continuous health plan enrolment (with medical and pharmacy benefits) for ≥12 months before and after index date. Patients were excluded if they had an AUC diagnosis code in the month before the index episode, had evidence of complicated cystitis, were pregnant, and/or had medications or urological procedures associated with chronic urologic conditions on index or during the baseline period. Detailed inclusion and exclusion criteria are provided in Table S1.

### Cohort stratification

For analysis purposes, the study population was stratified into separate cohorts as follows: (i) TF versus no TF; (ii) a history of AUC recurrence in the baseline period (pre-index recurrence) versus patients without prior AUC; and (iii) age (≤50 years versus >50 years at index), using age >50 years as a proxy for menopausal status, as guidelines in Japan vary based on menopausal status. Stratification by age was applied for analyses of treatment patterns and prevalence, while stratification by TF and pre-index recurrence status were used for all analyses.

### Outcome variables

Baseline demographics and clinical characteristics [age, Charlson Comorbidity Index (CCI) score, and top 10 comorbid conditions] were evaluated in the 12-month pre-index period. Prevalence of AUC (2016–2021) was calculated across all patients, and by age cohorts (pre-menopausal: ≥18 to <50 years and post-menopausal: ≥50 to <75 years). Annual AUC prevalence was calculated per 100 000 women. The annual rate of multiple AUC episodes was calculated as the number of patients with an index AUC diagnosis divided by the number of patients with ≥2 AUC diagnoses in each year of interest. Age-standardized annual prevalence data were estimated using published annual Japan census data.^18^ Briefly, for patients aged 18 to 74, prevalence was determined in age increments of 1 year, while prevalence for the population aged 75 years and over was taken as the mean prevalence for patients aged 70–74 years. These prevalence data in the JMDC database were multiplied by the size of the Japanese population for each age to arrive at an estimated number of actual patients with AUC in Japan for each age and overall, for each year of the study. AUC recurrence was defined as ≥2 AUC episodes in 6 months or ≥3 AUC episodes in 12 months, and the mean number of episodes per year (after the index episode) was reported, including additional AUC episodes. Post-index outcome variables included treatment patterns, TF, pre-index recurrence, and AUC-related and all-cause costs, and were reported for the full 12-month follow-up period. All baseline and outcome variables were reported for the overall population and by cohort (TF and pre-index recurrence).

All outcomes, stratified according to study cohorts, were evaluated in the follow-up period per AUC episode. Treatment patterns including initial (index antibiotics) versus subsequent treatments were determined. Per-patient AUC-related costs, per AUC index episode, and in the follow-up period, were calculated based on: inpatient care (hospital stay), outpatient care [ER visits, outpatient hospital visits, physician office visits (general practice), lab/imaging tests, home health visits and durable medical equipment], pharmacy costs (prescription costs), and total costs. Costs were adjusted to 2022 Japanese Yen.

### Statistical and multivariable analysis

Descriptive statistics are presented as mean and standard deviation (SD) for continuous variables; categorical and binary variables are presented as counts and percentages. Each outcome evaluated over different time-periods or separately between cohorts (including costs per episode, the total costs in 12-months, and differences across the cohorts) was compared using the student’s *t*-test or Wilcoxon rank sum test for continuous variables and chi-square test or Fisher’s exact test for categorical variables. All analysis were carried out using SAS 9.3/9.4 (Cary, NC).

Multivariable analyses were performed to evaluate the impact of recurrence and TF, per episode and during the 12-month period, using generalized linear models. See supplemental methods.

### Ethical considerations

This study complied with all applicable laws regarding participant privacy. No direct participant contact or primary collection of individual participant data occurred, and study results are tabulated and presented as aggregate analyses. JMDC provided a written oath to use their data for the study objectives. Informed consent, ethics committee, or institutional review board approval was not required.

## Results

### Demographic and clinical characteristics

Overall, 71 476 patients were included and the mean (SD) age was 43.88 (13.24) years, with 62.46% of patients aged <50 years. The overall population had a mean CCI (SD) of 0.77 (1.38), 5.24% (*n* = 3742) patients had evidence of TF and 4.49% (*n* = 3206) patients had pre-index recurrence. Patients with evidence of TF and those who experienced pre-index recurrence had a mean CCI (SD) of 0.93 (1.51) and 0.91 (1.51), respectively. For the overall population, the most common comorbidity was allergic rhinitis (31.25%), followed by astigmatism (25.85%). Similar characteristics were seen across TF and pre-index recurrence cohorts (Table 1).

**Table 1.** Baseline patient demographic and clinical characteristics

	All patients	TF status		Pre-index recurrence status	
		Evidence of TF	No evidence of TF	Evidence of pre-index recurrence	No evidence of pre-index recurrence
*N* (%)	71 476	3742 (5.24)	67 734 (94.76)	3206 (4.49)	68 270 (95.51)
Age at index date					
Age, mean (SD)	43.88 (13.24)	44.43 (13.16)	43.85 (13.24)	44.99 (13.66)	43.83 (13.22)
Age group, *n* (%)					
18–29	13 211 (18.48)	652 (17.42)	12 559 (18.54)	571 (17.81)	12 640 (18.51)
30–39	12 190 (17.05)	621 (16.60)	11 569 (17.08)	556 (17.34)	11 634 (17.04)
40–49	19 240 (26.92)	996 (26.62)	18 244 (26.93)	717 (22.36)	18 523 (27.13)
50–59	18 717 (26.19)	1047 (27.98)	17 670 (26.09)	891 (27.79)	17 826 (26.11)
60–69	6984 (9.77)	366 (9.78)	6618 (9.77)	394 (12.29)	6590 (9.65)
70+	1134 (1.59)	60 (1.60)	1074 (1.59)	77 (2.40)	1057 (1.55)
<50	44 641 (62.46)	2269 (60.64)	42 372 (62.56)	1844 (57.52)	42 797 (62.69)
50+	26 835 (37.54)	1473 (39.36)	25 362 (37.44)	1362 (42.48)	25 473 (37.31)
Baseline CCI					
CCI, mean (SD)	0.77 (1.38)	0.93 (1.51)	0.71 (1.34)	0.91 (1.51)	0.76 (1.37)
CCI score, *n* (%)					
0	51 555 (72.13)	2458 (65.69)	49 097 (72.49)	2163 (67.47)	49 392 (72.35)
1	1096 (1.53)	57 (1.52)	1039 (1.53)	49 (1.53)	1047 (1.53)
2	13 468 (18.84)	835 (22.31)	12 633 (18.65)	655 (20.43)	12 813 (18.77)
3	990 (1.39)	75 (2.00)	915 (1.35)	53 (1.65)	937 (1.37)
4+	4367 (6.11)	317 (8.47)	4050 (5.98)	286 (8.92)	4081 (5.98)
Top 10 comorbidities, *n* (%)					
Allergic rhinitis, unspecified	22 337 (31.25)	1418 (37.89)	20 919 (30.88)	1224 (38.18)	21 113 (30.93)
Astigmatism	18 479 (25.85)	1017 (27.18)	17 462 (25.78)	883 (27.54)	17 596 (25.77)
Dermatitis, unspecified	13 047 (18.25)	853 (22.8)	12 194 (18)	728 (22.71)	12 319 (18.04)
Chronic gastritis, unspecified	10 064 (14.08)	660 (17.64)	9404 (13.88)	616 (19.21)	9448 (13.84)
Low back pain	8975 (12.56)	591 (15.79)	8384 (12.38)	592 (18.47)	8383 (12.28)
Hyperlipidaemia, unspecified	8716 (12.19)	500 (13.36)	8216 (12.13)	476 (14.85)	8240 (12.07)
Essential (primary) hypertension	8311 (11.63)	472 (12.61)	7839 (11.57)	503 (15.69)	7808 (11.44)
Gastro-oesophageal reflux disease with oesophagitis	8026 (11.23)	533 (14.24)	7493 (11.06)	473 (14.75)	7553 (11.06)
Constipation	7751 (10.84)	541 (14.46)	7210 (10.64)	495 (15.44)	7256 (10.63)
Disorders of initiating and maintaining sleep (insomnias)	5942 (8.31)	395 (10.56)	5547 (8.19)	390 (12.16)	5552 (8.13)

CCI, Charlson Comorbidity Index; SD, standard deviation; TF, treatment failure.

### Prevalence rates

**Figure 1.**
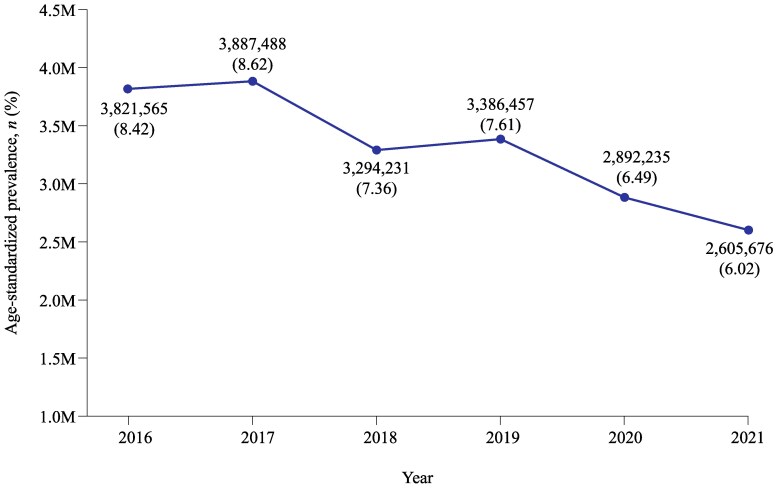
Age-standardized prevalence of AUC for adult women in Japan (2016–2021). Calculated by multiplying the mean number of AUC claims per woman in the JMDC dataset by the number of women (≥18 years) in Japan (census data). JMDC, Japanese Medical Data Centre; M, million.

The age-standardized prevalence of AUC in Japan (2016–2021) decreased over time (from 8.62% to 6.02%), with ∼2.6–3.9 million women affected each year. Estimated annual prevalence of AUC was lower in 2020 and 2021, compared with previous years (2016–2019; Figure 1). Overall, there were 142 026 patients with pre-index recurrence in Japan between the years 2016–2021. Among patients who experienced pre-index recurrence, the mean number of episodes per year was 3.45 (index AUC episode + 2.45 recurrent episodes; Table 2).

**Table 2.** Annual number of AUC episodes after index, among patients with pre-index recurrence

Total population with recurrent AUC	2016–2021^a,b^	2016	2017	2018	2019	2020	2021
All ages (18–75 years), *n*	142 026	11 250	18 614	23 644	28 471	29 851	29 687
AUC episodes after index, mean (SD)	2.45 (2.22)	2.36 (1.99)	2.45 (2.19)	2.45 (2.24)	2.47 (2.29)	2.48 (2.31)	2.44 (2.18)
Aged 18–49 years, *n*	80 613	6212	10 403	13 404	16 326	17 104	16 881
AUC episodes after index, mean (SD)	2.28 (2.10)	2.21 (1.91)	2.28 (2.05)	2.28 (2.10)	2.30 (2.15)	2.30 (2.17)	2.28 (2.07)
Aged 50–74 years, *n*	61 413	5038	8211	10 240	12 145	12 747	12 806
AUC episodes after index, mean (SD)	2.67 (2.36)	2.55 (2.07)	2.67 (2.22)	2.68 (2.39)	2.70 (2.45)	2.73 (2.46)	2.66 (2.31)

Annual mean number of AUC episodes after the first AUC diagnosis (index AUC diagnosis) among female population was calculated and was stratified by pre- versus post-menopause. An index AUC diagnosis was defined as the first AUC diagnosis to meet the following conditions per year of interest: (i) AUC diagnosis occurred in an outpatient setting and (ii) there was no prior claim related to an AUC diagnosis within 1 month.

SD, standard deviation.

^a^Included patient data were from 1 October 2015.

^b^Values estimated based on data from the first 11 months of 2021.

### Antibiotic treatment patterns

In the overall treatment population, the most common oral antibiotics prescribed at the index episode were FQs (59.49%) followed by third generation cephalosporins (34.27%). Among patients with evidence of TF, 45.48% received FQs as their index antibiotic, and 43.37% received third generation cephalosporins. Among patients without evidence of TF, 60.27% received FQs and 33.77% received third generation cephalosporins as their index antibiotic (Table 3).

**Table 3.** Antibiotic treatment patterns

	Overall	Age			TF status			Pre-index recurrence status		
	All patients	Pre-menopausal (<50 years)	Post-menopausal (≥50 years)	*P*-value	Evidence of TF	No evidence of TF	*P*-value	Evidence of pre-index recurrence	No evidence of pre-index recurrence	*P*-value
*N* (%)	71 476	44 641 (62.00)	26 835 (38.00)	—	3742 (5.00)	67 734 (95.00)	—	3206 (4.00)	68 270 (96.00)	—
Index antibiotic by class, *n* (%)										
Penicillin-combinations	604 (0.85)	375 (0.84)	229 (0.85)	0.85	71 (1.90)	533 (0.79)	<0.001	34 (1.06)	570 (0.83)	0.173
Second generation cephalosporins	2202 (3.08)	1515 (3.39)	687 (2.56)	<0.001	147 (3.93)	2055 (3.03)	<0.001	76 (2.37)	2126 (3.11)	0.017
Third generation cephalosporins	24 497 (34.27)	16 029 (35.91)	8468 (31.56)	<0.001	1623 (43.37)	22 874 (33.77)	<0.001	1056 (32.94)	23 441 (34.34)	0.103
Penems	717 (1.00)	449 (1.01)	268 (1.00)	0.93	94 (2.51)	623 (0.92)	<0.001	47 (1.47)	670 (0.98)	0.007
FQs	42 523 (59.49)	25 678 (57.52)	16 845 (62.77)	<0.001	1702 (45.48)	40 821 (60.27)	<0.001	1938 (60.45)	40 585 (59.45)	0.259
Fosfomycin	933 (1.31)	595 (1.33)	338 (1.26)	0.40	105 (2.81)	828 (1.22)	<0.001	55 (1.72)	878 (1.29)	0.036
Index antibiotic dose and days’ supply, mean (SD)										
Average daily dose	1.97 (1.00)	1.99 (1.00)	1.93 (0.99)	<0.001	2.24 (1.03)	1.95 (1.00)	<0.001	1.97 (1.01)	1.97 (1.00)	0.931
Average duration of treatment	5.13 (1.6)	5.08 (1.56)	5.21 (1.67)	<0.001	5.03 (1.67)	5.14 (1.60)	<0.001	5.37 (1.96)	5.12 (1.58)	<0.001
IV carbapenem, *n* (%)										
Carbapenem use in index episode	8 (0.01)	5 (0.01)	3 (0.01)	1.00	8 (0)	0 (0.00)	<0.001	0 (0)	8 (0.01)	1.000
Any IV carbapenem use in follow-up	97 (0.14)	66 (0.15)	31 (0.12)	0.26	17 (0.45)	80 (0.12)	<0.001	7 (0.22)	90 (0.13)	0.193
Prevalence of AUC recurrence in follow-up period, *n* (%)										
Patients with ≥1 AUC diagnoses within 6 mo. of index (after index episode)	6485 (9.07)	3624 (8.12)	2861 (10.66)	<0.001	396 (10.58)	6079 (8.97)	<0.001	645 (20.12)	5840 (8.55)	<0.001
Patients with ≥2 AUC diagnoses within 12 mo. of index	2638 (3.69)	1333 (2.99)	1305 (4.86)	<0.001	212 (5.67)	2427 (3.58)	<0.001	393 (12.26)	2245 (3.29)	<0.001
Patients with either ≥1 AUC in 6 mo. or ≥2 AUC in 12 mo.	7139 (9.99)	3947 (8.84)	3192 (11.89)	<0.001	465 (12.43)	6665 (9.84)	<0.001	738 (23.02)	6401 (9.38)	<0.001
AUC episodes in follow-up period, *n* (%)										
Number of AUC episodes per patient(index + follow-up)	1.25 (0.6)	1.22 (0.55)	1.3 (0.67)	<0.001	1.34 (1.00)	1.24 (0.60)	<0.001	1.61 (1.00)	1.23 (0.57)	<0.001
Number of patients with ≥1 follow-up AUC episode after index episode	13 310 (18.62)	7422 (16.63)	5888 (21.94)	<0.001	900 (24.05)	12 402 (18.31)	<0.001	1208 (37.68)	12 102 (17.73)	<0.001
Number of follow-up episodes per patient (12-mo. follow-up) among patients with ≥1 follow-up episodes	1.34 (0.7)	1.31 (0.65)	1.38 (0.75)	<0.001	1.42 (1.00)	1.33 (0.69)	<0.001	1.63 (1.00)	1.31 (0.65)	<0.001
Number of follow-up episodes per patient (12-mo. follow-up) among all patients	1.25 (0.6)	1.22 (0.55)	1.3 (0.67)	<0.001	1.34 (1.00)	1.24 (0.60)	<0.001	1.61 (1.00)	1.23 (0.57)	<0.001
Prevalence of antibiotic TF, *n* (%)										
The proportion of patients who received a second oral antibiotic, IV antibiotic (following their initial treatment), or a second diagnosis of AUC in an acute care setting (ED or inpatient) during their index AUC episode	3742 (5.24)	2269 (5.08)	1473 (5.49)	0.02	3742 (100)	—	—	243 (7.58)	3499 (5.13)	<0.001
Time to TF, mean days (SD)										
Mean days to TF	10.33 (7.33)	10.5 (7.43)	10.06 (7.16)	0.07	10.33 (7.33)	—	—	11.65 (7.25)	10.23 (7.33)	<0.001
Time to TF (evidence of 2nd oral treatment, *n* (%)										
Penicillin-combinations	97 (0.14)	56 (0.13)	41 (0.15)	0.34	97 (2.59)	—	—	6 (0.19)	91 (0.13)	0.42
Second generation cephalosporins	93 (0.13)	58 (0.13)	35 (0.13)	0.99	93 (2.49)	—	—	5 (0.16)	88 (0.13)	0.61
Third generation cephalosporins	1388 (1.94)	860 (1.93)	528 (1.97)	0.70	1388 (37.09)	—	—	102 (3.18)	1286 (1.88)	<0.001
Penems	184 (0.26)	116 (0.26)	68 (0.25)	0.87	184 (4.92)	—	—	10 (0.31)	174 (0.25)	0.53
FQs	1535 (2.15)	902 (2.02)	633 (2.36)	<0.001	1535 (41.02)	—	—	97 (3.03)	1438 (2.11)	<0.001
Fosfomycin	170 (0.24)	105 (0.24)	65 (0.24)	0.85	170 (4.54)	—	—	11 (0.34)	159 (0.23)	0.21
Evidence of IV AB	275 (0.38)	172 (0.39)	103 (0.38)	0.98	275 (7.35)	—	—	12 (0.37)	263 (0.39)	0.92
Multiple TF on index episode *n* (%)										
Patients with 2 TFs	376 (0.53)	203 (0.45)	173 (0.64)	<0.001	376 (10.05)	—	—	30 (0.94)	346 (0.51)	<0.001
Patients with ≥3 TFs	145 (0.20)	76 (0.17)	69 (0.26)	0.01	145 (3.87)	—	—	16 (0.50)	129 (0.19)	<0.001

AB, antibiotic; ED, emergency department; FQ, fluoroquinolone; IV, intravenous; mo., months; SD, standard deviation; TF, treatment failure.

During the follow-up period, AUC recurrence was documented in 9.99% of patients overall. Among patients with and without evidence of TF, the rates of recurrence were 12.43% and 9.84%, respectively (*P* < 0.001). Among patients with pre-index recurrence, 23.02% experienced recurrence in the post-index follow-up period, compared with 9.38% of patients without evidence of pre-index recurrence (*P* < 0.001). Furthermore, 37.68% of patients with evidence of pre-index recurrence had ≥1 AUC episode during follow-up, with a mean number of 1.63 follow-up episodes per patient. TF was higher in patients with evidence of pre-index recurrence (7.58%) compared with patients without pre-index recurrence (5.13%; *P* < 0.001; Table 3).

When analyzed by age (<50 and ≥50 years as a proxy for menopausal status), FQs were prescribed to 57.52% of pre-menopausal women and 62.77% of post-menopausal women. Prevalence of recurrence during the follow-up period was higher among post-menopausal women compared with pre-menopausal women (11.89% versus 8.84%; *P* < 0.001; Table 3).

**Figure 2.**
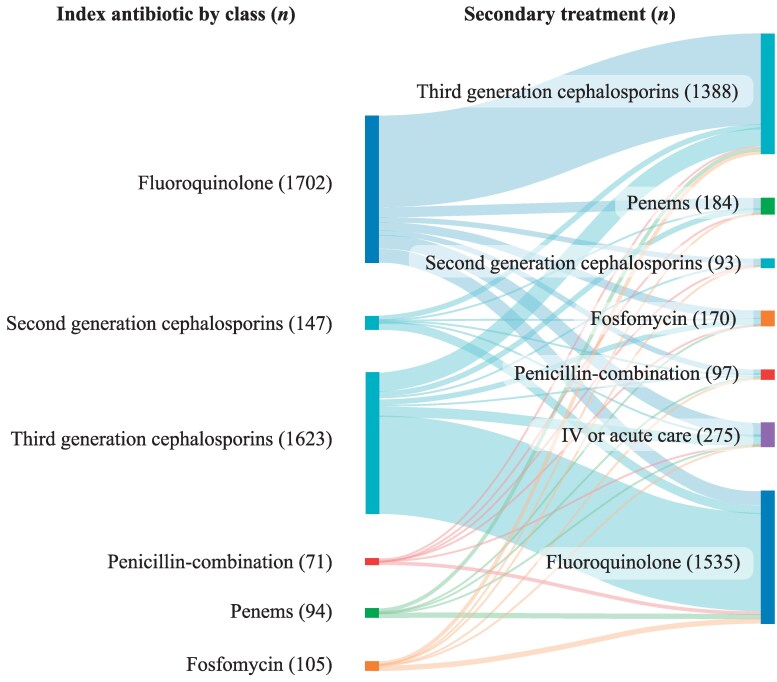
Sankey diagram of antibiotic TF. Patients with evidence of TF (*N* = 3742). IV, intravenous; TF, treatment failure.

Of the 3742 patients with evidence of TF, 367 (9.81%) patients received the same class of drug as their index antibiotic following TF. FQs and third generation cephalosporins were the main antibiotics prescribed as both index and secondary treatment. A small proportion of patients received IV antibiotics or acute care (*n* = 275; 7.35%) as secondary treatment (Figure 2).

### AUC-related and all-cause costs for index AUC episode

**Figure 3.**
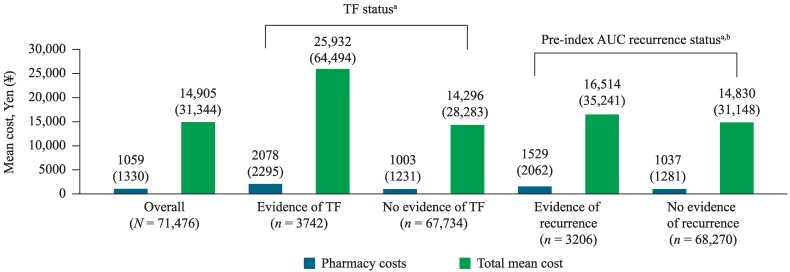
Per-patient AUC-related costs (JPY) for index AUC episode. ^a^Pharmacy and total costs: all *P* < 0.001. ^b^Total costs: *P* = 0.008. Pharmacy costs in USD ($), mean (SD). Overall pharmacy costs: $7.71 ($9.68); pharmacy costs for evidence of TF: $15.12 ($16.70); pharmacy costs for no evidence of TF: $7.30 ($8.96); pharmacy costs for evidence of recurrence: $11.13 ($15.00); pharmacy costs for no evidence of recurrence: $7.55 ($9.32). Total costs (inpatients, outpatients and pharmacy) in USD ($), mean (SD). Overall costs: $108.47 ($228.09); total costs for evidence of TF: $188.71 ($469.32); total costs for no evidence of TF: $104.03 ($205.82); total costs for evidence of recurrence $120.17 ($256.45); total costs for no evidence of recurrence: $107.92 ($226.66). JPY, Japanese Yen; SD, standard deviation; TF, treatment failure; USD, United States dollar.

Overall, the mean total AUC-related costs (SD) were ¥14 905 (¥31 344) and pharmacy costs were ¥1059 (¥1330) per patient, per index AUC episode. Patients with evidence of TF had significantly higher mean (SD) pharmacy [¥2078 (¥2295)] and total costs [¥25 932 (¥64 494)] per patient versus patients without evidence of TF (*P* < 0.001). Patients with pre-index recurrence had higher mean (SD) pharmacy [¥1529 (¥2062)] and total costs [¥16 514 (¥35 241)] per patient per index episode versus patients without pre-index recurrence who incurred total costs of ¥14 830 (¥31 148; *P* = 0.008; Figure 3).

For all-cause costs per patient per index AUC episode, the mean (SD) pharmacy costs for patients with evidence of TF [¥14 763 (¥79 903)] were approximately twice those without TF [¥7170 (¥52 958); *P* < 0.001]. The total mean (SD) costs for patients with evidence of TF [¥81 494 (¥226 557)] were significantly higher than those without TF [¥33 743 (¥68 577); *P* < 0.001]. Patients with pre-index recurrence had significantly higher mean (SD) pharmacy costs [¥13 136 (¥80 934)] and total costs [¥42 517 (¥75 762)] versus patients without pre-index recurrence [¥7306 (¥53 164) and ¥35 948 (¥85 589), respectively; *P* < 0.001; Figure S2].

### Multivariable cost model—TF versus no TF and pre-index recurrence versus no pre-index recurrence

After adjusting for pre-index recurrence at baseline, age, CCI score, pharmacy claims, total costs, and index antibiotic class, patients with TF had significantly higher total and pharmacy costs than patients without TF (*P* < 0.001; Table S2). On average, costs were 3.59 times higher for pharmacy costs and 1.74 times higher for total costs in the TF cohort during the index episode. When the index episode and the 12-months follow-up were considered, costs were also significantly higher for pharmacy costs (2.69 times higher) and total costs (1.53 times higher) for patients with TF than for patients with no evidence of TF during the index episode (*P* < 0.001; Table S2). The results of the multivariable model for pre-index recurrence versus no pre-index recurrence for cost indicated that during the index episode and 12-months follow-up period, costs were significantly higher for pharmacy costs (1.82 times higher) and total costs (1.37 times higher) for patients with pre-index recurrence versus no pre-index recurrence (*P* < 0.001; Table S3).

## Discussion

In this retrospective study of claims data in Japan, over 60% of patients with AUC were aged <50 years, and the proportions of patients who experienced TF and pre-index recurrence were 5.24% and 4.49%, respectively. AUC recurrence reported in this study correlates with another study, which reported a similar overall recurrence rate of 4.1% and 4.4% in female patients alone.^8^ In this study, there were higher prevalence rates among women aged ≥50 years, although there was a notable drop in overall prevalence in the years 2020 and 2021. Most patients received FQs as their index antibiotic, in line with treatment guidelines. Economic burden of AUC was greatest in patients who had evidence of TF or in those who had pre-index recurrence.

There are limited studies reporting AUC prevalence between 2016 and 2021 in Japan. Based on a global analysis of UTI incidence, there were over 404.6 million individuals with UTI globally in 2019, suggesting a rising trend of UTI burden between 1990 and 2019.^19^ In this study, the estimated annual prevalence of AUC decreased from 8.62% to 6.02% (2016–2021), with ∼2.6–3.9 million women affected each year. We propose that the drop (from 6.49% to 6.02%) in estimated annual prevalence seen in this study between 2020 and 2021 may have been due to the reduced number of patients seeking in-person treatment for mild infections during the SARS-CoV2 pandemic in those years. Further analyses are needed in the post-acute SARS-CoV2 period to observe the prevalence of AUC in Japan. While AUC prevalence rates showed a slight decreasing trend over the period 2016–2021, the number of patients with pre-index AUC recurrence did not decrease during this period but rather increased (the size of the database population remined largely constant over the study period). Of note, however, the mean number of post-index AUC episodes among patients with pre-index recurrence remained stable across the study period.

Results of this analysis (2016–2021) show that prescribing patterns in Japan are following the 2015 JAID/JSC guidelines for FQs and third generation cephalosporins.^9^ Across all cohorts evaluated, FQs and third generation cephalosporins were the most commonly prescribed antibiotics, accounting for ∼95% of all prescriptions. These data correlate with a retrospective study by Takahashi *et al*.,^8^ which also reported that FQs and third generation cephalosporins were prescribed most commonly to patients with uncomplicated cystitis in Japan, albeit at a lower frequency than in the current study (FQs and third generation cephalosporins were prescribed to 30.4% and 31.4% of female patients, respectively).^8^ The lower frequency of antibiotic prescriptions is likely due to the different patient populations and data sets. Takahashi *et al*.,^8^ included elderly individuals from six medical institutions with a median age of 71 years and 81% were over 50 years, whereas the mean age for the current study was 43.88 years; the data analyzed also included claims data from inpatient, outpatient, and pharmacy settings. Additionally, an observational study of claims data from Japan by Kusama *et al*.,^15^ reported prescriptions of FQs and cephalosporins in ∼90% of patients with uncomplicated cystitis (FQ, 52.67%; cephalosporins, 36.95%).^15^ Although prescribing patterns in the current study align with treatment guidelines,^9^ a Japanese surveillance study by Wada *et al*.,^11^ on antimicrobial susceptibility of bacterial pathogens in patients with AUC, found increased rates of FQ-resistant and ESBL-producing *E. coli* compared with prior nationwide surveys.^11,20^ In particular, there were significantly higher rates of FQ-resistant and ESBL-producing *E. coli* in post-menopausal women. Overall, the authors concluded that the inappropriate use of FQs and third generation cephalosporins had led to this increased resistance and should be avoided as first choice for AUC treatment.^11,20^ Furthermore, the European Medicines Agency and the United States Food and Drug Administration have restricted FQ use due to their long-lasting and potentially irreversible adverse events.^21,22^ This pattern of FQ and third generation cephalosporins use in Japan could also potentially pose problems for future AMR strategies. In particular, the National Action Plan (NAP) on AMR set a target to lower the rates of FQ-resistant *E. coli* to 25% or less by 2020;^22^ however, the target percentage of drug-resistant bacteria has not been reached, with reported resistance rate to FQ in *E. coli* of 41.5% in 2020.^23^ As a result, the NAP on AMR 2023 has set another goal to maintain FQ resistance of *E. coli* at 30% or less by 2027, and reduce the use of third generation cephalosporins and FQs per day per 1000 inhabitants by 40% and 30%, respectively, from 2020 levels by 2027.^24^

The most recent JAID/JSC treatment guidelines for antimicrobial use were published in 2023 (Japanese language only), after the treatment period (2016–2021) examined in this study.^25^ Improvements in treatment outcomes and healthcare costs are expected as a result of the country’s ongoing antimicrobial stewardship efforts. Since the implementation of nationwide strategies in 2016, including placing restrictions on the use of quinolones and third generation cephalosporins, there has been notable suppression of multidrug-resistant organisms.^24^ If this trend continues, supported by the most recent treatment guidelines for antimicrobial use, improvements in the success rates of first-line antibiotic treatment can be expected. This will be accompanied by a reduced need for follow-up visits and alternative antibiotic treatments and fewer complications and hospitalizations, which may in turn equate to significant savings in healthcare costs.

Sulfamethoxazole-trimethoprim and fosfomycin are not typically used as first-line treatments for AUC caused by Gram-positive organisms as their activity is limited to certain strains.^26,27^ These antibiotics do not provide adequate coverage against *Staphylococcus saprophyticus* or *Enterococcus* spp., which are important Gram-positive uropathogens causing AUC in pre-menopausal women.^27,28^ In Japan, resistance to sulfamethoxazole-trimethoprim is relatively high.^29^ The use of fosfomycin is much restricted due to the unavailability of susceptibility data—fosfomycin is not typically tested in surveillance studies and requires manual susceptibility testing due to its omission from automated susceptibility panels.^30^ Given that AUC treatment is typically empiric, clinicians tend to prioritize antibiotics with broader coverage against all uropathogens to reduce the risk of TF.^8^ There is the prospect that new treatment options may become available in Japan in the near future with the recent confirmation of clinical efficacy in phase III clinical evaluation of a novel, first-in-class triazaacenaphthylene antibiotic for the treatment of AUC^31^ that in March 2025 received approval for the same indication in the United States.^32^

In our study, patients who experienced TF were largely switched to another class of antibiotic, indicating prescribers were considering alternative AUC treatment options, but only after TF. TF was associated with increased economic burden in our study, with increased rates of AUC recurrence in the post-index follow-up period and increased costs, with both pharmacy (¥2078) and total costs (¥25 932) reported as significantly higher for patients with TF compared with patients without TF on the index episode. Similarly, patients with evidence of pre-index recurrence had both higher pharmacy (¥1529) and total costs (¥16 514) per patient compared with patients without pre-index recurrence. Based on surveillance data indicating an increase in the proportion of drug-resistant bacteria, and given that some studies identify drug resistance as a risk factor for AUC recurrence,^33–35^ it is possible that the increase in drug-resistant bacteria such as *E. coli* has contributed to the burden of TF and recurrence.

### Limitations

In this claims data analysis, the following limitations should be considered. The index date was anchored to the prescription date for the index oral antibiotic, but it was not possible to tie the medical diagnosis claim of AUC to the exact prescription date in every instance. Since the antibiotic prescription and diagnosis had to occur within the same month, some patients were excluded from our analysis if their prescription date was too close to the beginning or end of the month. As such, it is possible that we excluded patients with AUC with an oral antibiotic prescription if recorded in a different month from the diagnosis.

Another limitation was the inability to separate cost data into ‘traditional’ claims analyses categories (e.g. inpatient, outpatient, pharmacy, and total costs), meaning we were limited to reporting pharmacy costs and total costs. Additionally, our definition of UTI recurrence differed from other previously published literature.^3,4^ We defined recurrence as two AUC episodes within 6 months, or three AUC episodes within 1 year, where the episodes must be at least 2 months apart, given the lack of exact diagnosis dates in the JMDC data. Furthermore, as a retrospective study, these data can only demonstrate association and not causality, and the results of this study may not be generalizable to the entire female AUC population in Japan. Lastly, the JMDC database mainly covers a pre-retirement population of individuals who are <75 years of age and the results might not be generalizable to the entire post-menopausal population.

A further limitation of this database study is that the diagnosis of AUC was based on clinical ICD-10 codes; the absence of clinical data describing patient symptoms may have led to potential disease misclassification in cases where the incorrect diagnostic codes were used. It was assumed that if a diagnostic code for AUC was recorded in the database alongside documented antibiotic treatment, then the patient was symptomatic according to clinical guidelines. While there is a possibility that in some cases asymptomatic bacteriuria may have been miscoded as AUC and treated with antibiotics, such misclassification would be expected to be non-differential and affect all analysis groups essentially equally. Moreover, it is unlikely that patients without urinary symptoms would be prescribed antibiotics when treatment aligns to Japanese guidelines. Therefore, asymptomatic bacteriuria is not thought to be a major source of bias in this study.

Although overall costs were modest, healthcare providers should consider the cost implications for patients with AUC TF and the history of recurrence when selecting antibiotics for empiric treatment. This should include thorough verification of medical history at the time of diagnosis, selection of drugs based on susceptibility testing, and the development of new medications that are effective against drug-resistant bacteria.

In this real-word study, the prevalence of AUC between 2016 and 2021 in Japan is described, with up to 3.9 million women affected each year. Antibiotic treatments were consistent with guidelines. Patients who experienced TF or AUC recurrence had significantly higher overall healthcare related costs, indicating an increased economic burden associated with managing these patients.

## Supplementary Material

dlaf178_Supplementary_Data

## Data Availability

Data used for this publication was generated by JMDC. For access to anonymized subject level data, please contact JMDC.

## Supplementary data

Figures S1 and S2 and Tables S1 to S3 are available as Supplementary data at *JAC-AMR* Online.
