# Supplementary material for: Prevalence and economic evaluation of acute uncomplicated cystitis in women from Japan: a retrospective cohort study
Source: JAC Antimicrob Resist. 2025 Oct 17;7(5):dlaf178. doi: 10.1093/jacamr/dlaf178 (PMC12531626; doi:10.1093/jacamr/dlaf178)
Supplement: dlaf178_Supplementary_Data [file dlaf178_supplementary_data.docx]

**Supplementary materials**

***Materials and methods***

***Study design***

Please see the main article for more details on the study design and objectives.

While the Japanese Medical Database Center uses International Classification of Diseases 10^th^ Revision, Clinical Modification codes, Current Procedural Terminology or National Drug Codes are not used in Japan; thus, a crosswalk to the Japanese classification system for Current Procedural Terminology and National Drug Codes was implemented.

***Multivariable analysis***

Patient demographics (age, geographic region), clinical characteristics (recurrences, Charlson Comorbidity Index score), baseline total costs, and index treatment drug class were controlled and adjusted in the model. The dependent variables were AUC-related costs and total healthcare costs, and the primary independent variables for one analysis was treatment failure (TF), and recurrence for the second analysis. For cost outcomes, gamma distribution and log link were used. Marginal effect with 95% confidence interval and p-values were calculated to examine the differences between the two cohorts.

***Results***

**Summary of results (max: 47/50 words):** Figure S1 is an illustration of the study design. Figure S2 shows the all-cause costs for index AUC episode per patient. Table S1 includes the study definitions and inclusion/exclusion criteria. Multivariable cost model for TF and
pre-index recurrence status are shown in Tables S2 and S3, respectively.

**Figure S1.** Study design.

**
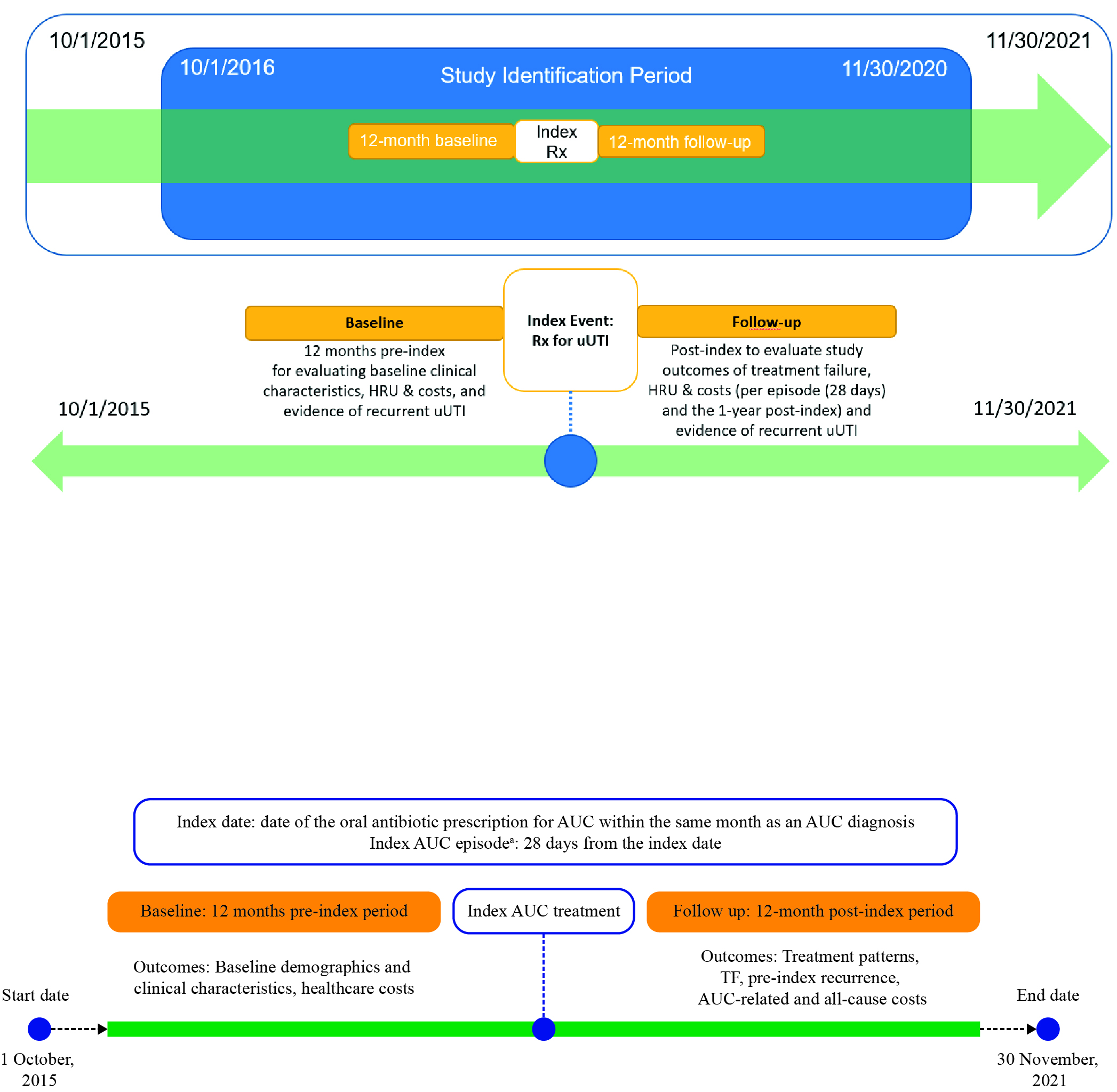
**

^a^If TF was observed, the episode was extended 28 days from the date of the observed TF.

TF, treatment failure.

## **Figure S2.** Per-patient all-cause costs (JPY) for index AUC episode**.**


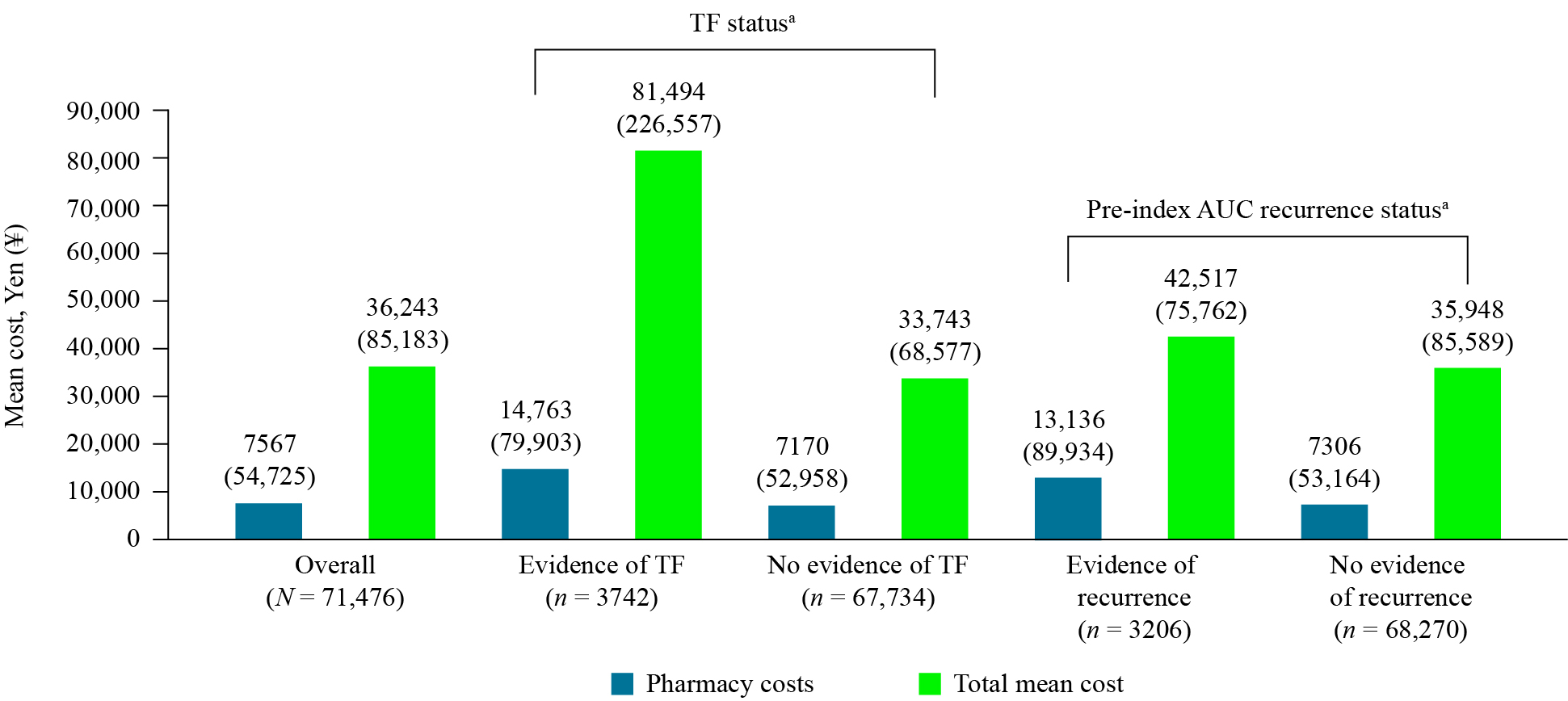


^a^Pharmacy and total costs: all *P*<0.001. Pharmacy costs in USD ($), mean (SD). Overall pharmacy costs: $55.07 ($398.23); pharmacy costs for evidence of TF: $107.43 ($581.46); pharmacy costs for no evidence of TF: $52.17 ($385.38); pharmacy costs for evidence of recurrence: $95.59 ($588.96); pharmacy costs for no evidence of recurrence: $53.16 ($386.87). Total costs (inpatients, outpatients and pharmacy) in USD ($), mean (SD). Overall costs: $263.74 ($619.88); total costs for evidence of TF: $593.03 ($1648.65); total costs for no evidence of TF: $245.55 ($499.03); total costs for evidence of recurrence $309.40 ($551.32); total costs for no evidence of recurrence: $261.60 ($622.83).

JPY, Japanese Yen; SD, standard deviation; TF, treatment failure; USD, United States Dollar.

**Table S1.** Study definitions and inclusion/exclusion criteria.

| **Term** | **Definition** |
| --- | --- |
| **TF^a^** | Patients had evidence of TF if they experienced any of the following:   - Received intravenous antibiotics within 28 days of the index date - Had a second oral antibiotic prescription claim (occurrence of a different antibiotic than the initial treatment) within 28 days of the index date - Had a second AUC diagnosis in an acute care setting (ER or hospital) within 28 days of the index date |
| **AUC recurrence^b^** | - ≥2 AUC episodes within 6 months or ≥3 AUC episodes within 12 months (including the index AUC) |
| **Index date** | - Date of the oral antibiotic prescription for an AUC within the same month of an AUC diagnosis that had ≥12 months continuous health plan enrolment before and after |
| **AUC episode** | - An acute episode of uUTI/uncomplicated cystitis, lasting up to 28 days from index date, pending no observed TF |
| **Index AUC episode** | - AUC episode was defined as 28 days starting from the index date |
| **Inclusion criteria** | Patients were included from the study population if they met the following:   - ≥18 years old on the index date - ≥1 oral antibiotic prescription claim with days’ supply of >0 days in the same calendar of the first observed AUC diagnosis during the study identification period - ≥1 diagnosis code claim with a primary or secondary diagnosis of uUTI (ICD-10-CM) in the same calendar month as the oral antibiotic prescription for the first observed AUC - ≥12 months continuous health plan enrolment with medical and pharmacy benefits prior to the index date and ≥12 months post index date |
| **Exclusion criteria^c^** | Patients were excluded from the study population if they had any of the following:   - Had evidence of complicated AUC for index AUC episode, defined as:   1) having received intravenous antibiotic treatment before the start of oral antibiotic therapy for AUC within ±5 days of the index date  2) had selected complicating comorbidities such as complicated or uncontrolled diabetes, cancer with immunosuppressants, severe CKD or ESRD on index or during baseline period  3) had selected complicating acute conditions, including pyelonephritis and kidney stones, during index month or 2 calendar months prior to index month   - Had medications or urological procedures associated with chronic urologic conditions on index or during baseline period - Had underlying urologic abnormalities (e.g. abnormality of the urinary tract, neurologic condition) on index or during baseline period - Had an inpatient admission within 3 months before the index date or within 2 days after the index date - Were pregnant on index or during the baseline period - Had an AUC diagnosis code in the month before the index episode |

^a^If TF was identified, the index episode was extended 28 days from the date of TF.

^b^Episodes were required to be ≥2 months apart given the inability to link an exact date to the medical claims and unknown diagnosis dates.

^c^This study included female patients only, as by definition, UTIs in male patients are considered complicated infections.

CKD, chronic kidney disease; ER, emergency room; ESRD, end-stage renal disease; ICD-10-CM, International Classification of Diseases, 10th Edition, Clinical Modification; TF, treatment failure; uUTI, uncomplicated urinary tract infection.

**Table S2.** Multivariable cost model – TF versus no TF.

|  | **TF status** | | **Average cost ratio** | ***P*-value** |
| --- | --- | --- | --- | --- |
|  | **TF** | **No TF** |  |  |
|  | **Average cost^a^**  **(95% CI)** | **Average cost^a^**  **(95% CI)** |  |  |
| **AUC-related cost: index episode, per patient** | | | | |
| Pharmacy costs | ¥2980.19  (¥2877.85–¥3086.16) | ¥829.87  (¥824.83–¥834.95) | 3.59 | <0.001 |
| Total costs | ¥23,788.32  (¥23,174.24–¥24,418.66) | ¥13,710.22  (¥13,639.65–¥13,781.15) | 1.74 | <0.001 |
| **AUC-related cost: index + follow-up episodes (12-moth), per patient** | | | | |
| Pharmacy costs | ¥3765.99  (¥3587.69–¥3953.15) | ¥1397.60  (¥1384.01–¥1411.32) | 2.69 | <0.001 |
| Total costs | ¥33,232.02  (¥32,116.07–¥34,386.76) | ¥21,735.28  (¥21,580.87–¥21,890.80) | 1.53 | <0.001 |

^a^Model controls for the following independent baseline variables: recurrences, age, CCI score, outpatient visit, pharmacy visit, total cost, index drug class along with TF status for recurrence model.

CCI, Charlson Comorbidity Index; CI, confidence interval; TF, treatment failure.

**Table S3.** Multivariable cost model – pre-index recurrence versus no pre-index recurrence.

|  | **Pre-index recurrence status** | | **Average cost ratio** | **p-value** |
| --- | --- | --- | --- | --- |
|  | **Pre-index recurrence** | **No pre-index recurrence** |  |  |
|  | **Average cost***  **(95% CI)** | **Average cost***  **(95% CI)** |  |  |
| **AUC-related cost: index episode, per patient** | | | | |
| Pharmacy costs | ¥1,151.67  (¥1,119.17–¥1,185.10) | ¥875.34  (¥869.40–¥881.31) | 1.32 | <0.001 |
| Total costs | ¥13,899.99  (¥13,584.12–¥14,223.21) | ¥14,210.39  (¥14,139.53–¥14,281.62) | 0.98 | 0.0657 |
| **AUC-related cost: index + follow-up episodes (12 months), per patient** | | | | |
| Pharmacy costs | ¥2,607.97  (¥2,486.04–¥2,735.87) | ¥1,430.46  (¥1,418.32–¥1,442.70) | 1.82 | <0.001 |
| Total costs | ¥29,537.75  (¥28,505.13–¥30,607.78) | ¥21,625.17  (¥21,477.94 – ¥21,773.41) | 1.37 | <0.001 |

*Model controls for the following independent baseline variables: recurrences, age, CCI score, outpatient visit, pharmacy visit, total cost, index drug class along with TF status for recurrence model.

CCI, Charlson Comorbidity Index; CI, confidence interval; TF, treatment failure.
